# Juvenile arthritis disease activity score is a better reflector of active disease than the disease activity score 28 in adults with polyarticular juvenile idiopathic arthritis

**DOI:** 10.1136/annrheumdis-2015-208462

**Published:** 2015-12-29

**Authors:** Qiong Wu, Hema Chaplin, Nicola Ambrose, Debajit Sen, Maria J Leandro, Charlotte Wing, Nicola Daly, Kate Webb, Corinne Fisher, Linda Suffield, Francesca Josephs, Clarissa Pilkington, Despina Eleftheriou, Muthana Al-Obaidi, Sandrine Compeyrot-Lacassagne, Lucy R Wedderburn, Yiannis Ioannou

**Affiliations:** 1 Arthritis Research UK Centre for Adolescent Rheumatology, University College London, London, UK; 2 Adolescent Rheumatology Department, University College London Hospital NHS Trust, London, UK; 3 Paediatric Rheumatology Department, Great Ormond Street Hospital for Children NHS Trust, London, UK; 4 Infection, Immunity, Inflammation, and Physiological Medicine Programme, Institute of Child Health, University College London, London, UK

**Keywords:** Juvenile Idiopathic Arthritis, DAS28, Rheumatoid Arthritis

A considerable proportion of children with polyarticular juvenile idiopathic arthritis (polyJIA) experience active disease into adulthood.^1^ However, there is no validated disease activity measure for adults with polyJIA, and they are often assessed using the disease activity score 28 (DAS28). DAS28 is validated in adults with rheumatoid arthritis (RA), and determines qualification for biological drugs in the UK and other countries.^2^ ^3^ In contrast to the juvenile arthritis disease activity score (JADAS),^4^ DAS28 does not fully evaluate the pattern of joint involvement often observed in polyJIA. In this study, we compared DAS28 with JADAS-10 in adolescents and adults with polyJIA.

Tender and swollen joint counts out of 28, active joint count of all joints up to a maximum of 10, patient/parent and physician global assessment visual analogue scales were collected from clinics in patients aged ≥10 years with polyJIA (International League of Associations for Rheumatology classification criteria for rheumatoid factor-negative (RhF-ve) or rheumatoid factor-positive (RhF+ve) polyJIA). Erythrocyte sedimentation rate (ESR) values were taken within 30 days before or after assessment. When unavailable, values were taken within 3 months before or after, provided the patient remained stable between the ESR test and assessment. When unavailable within these time periods, patients were excluded from analysis. DAS28 and JADAS-10 were calculated and compared using Spearman's rank correlation coefficient. DAS28>5.1 constitutes high-disease activity in adults with RA, ≤3.2 suggests low-disease activity or remission.^2^ ^3^ In children with polyJIA, JADAS-10>10.5 is considered to reflect high-disease activity,^5^ ≤3.8 reflects low or inactive disease.^6^ ^7^

**Figure 1:**
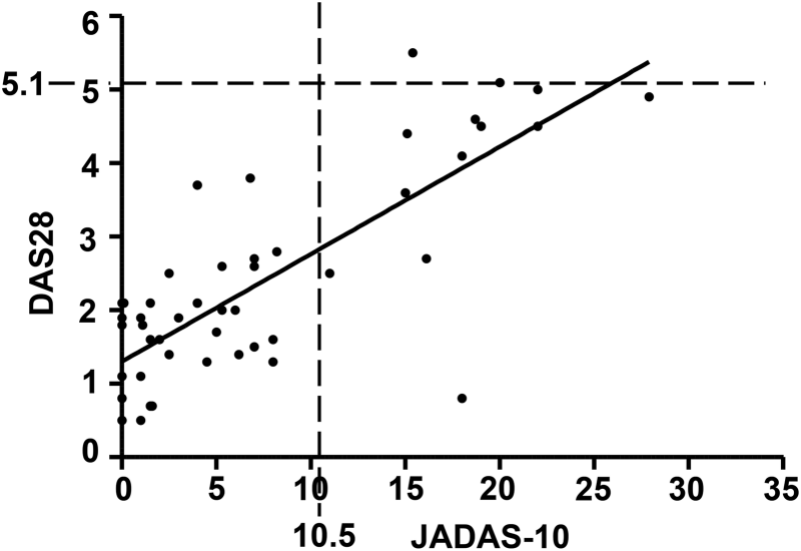
Scatter plot with linear regression line of juvenile arthritis disease activity score 10 (JADAS-10, x-axis) and disease activity score 28 (DAS28, y-axis). Despite good correlation between these disease activity measures, there is a discrepancy in the threshold for high-disease activity between JADAS-10 (JADAS-10 >10.5, vertical dotted line) and DAS28 (DAS28 >5.1, horizontal dotted line). Also, 13 out of 49 patients were classified as high-disease activity by JADAS-10 (data points to the right of the vertical dotted line), and of these, only 1 was defined as also being high-disease activity by DAS28 (data point above the horizontal dotted line). No patients were classified as high activity for DAS28 but not high according to JADAS-10.

Forty-nine patients (36 polyJIA-RhF-ve, 10 polyJIA-RhF+ve, 3 unknown RhF) were analysed (range=10–27 years, median=15 years, M:F ratio 1:3.5). Good correlation was seen between the disease activity scores (Spearman's r=0.69, p<0.0001). However, when looking at values above cut-offs defined for active disease, considerable underestimation of disease activity by DAS28 was observed with 13 patients having JADAS-10>10.5 but only 1 of these patients having DAS28>5.1 (figure 1).

There was no considerable difference in correlation between disease activity scores between adolescents (range=10–15 years, median=13 years, n=25, Spearman's r=0.83, p<0.0001) and adults (range=16–27 years, median=17 years, n=24, Spearman's r=0.73, p<0.0001), nor between polyJIA-RF-ve (n=36, Spearman's r=0.68, p<0.0001) and polyJIA-RF+ve (n=10, Spearman's r=0.80, p=0.0088).

Although previous studies found good correlations between DAS28 and JADAS in children with JIA,^8^ this is the first to include the adult population and highlight a discrepancy in thresholds for high-disease activity between DAS28 and JADAS.

Looking at individual patients, 9 out of 10 patients with active disease defined by JADAS (table 1) had joints deemed to be active that are not included in the DAS28 joint count, thus contributing to a high JADAS but not affecting DAS28. It would be pertinent to also calculate DAS44, but data were incomplete. If total active joints are greater than DAS28 swollen or tender joints, this could strongly imply JADAS incorporates joints outside those surveyed by DAS28. Seventy-five per cent of patients with active disease defined by JADAS but not DAS28 had higher total active joints than 28 tender or swollen joints compared with 17% of those patients not classified as active by JADAS and DAS28. This further suggests that a discrepancy in the proportion of patients with active disease as defined by JADAS and DAS28 respectively is predominantly due to differences in the number and distribution of joints surveyed.

**Table 1:** Joint counts in patients with active disease as defined by juvenile arthritis disease activity score-10

Patient code	Tender joints	Swollen joints	Active joints	RF status
13	**L ankle**	None	**L ankle, L subtalar**	Neg
17	R knee	R knee	R knee, **L ankle, R ankle, L subtalar,** **R subtalar, L midfoot, R midfoot**	Neg
30	**L TMJ, R TMJ**	L index MCP, R index MCP, L middle MCP, R middle MCP	**L TMJ, R TMJ,** 2 MCPs	Pos
31	Unknown	Unknown	Unknown	Neg
33	L wrist, R wrist, R middle MCP, L knee, R knee, **L ankle**, **R ankle**	L wrist, R wrist, R middle MCP, L knee, R knee, **L ankle**, **R ankle**	L wrist, R wrist, R middle MCP, L knee, R knee, **L ankle, R ankle**	Pos
36	6 MCPs, **L ankle**, **L subtalar**	L elbow, L wrist, R wrist, 6 MCPs, 6 PIPs, **L ankle, R ankle,** **L subtalar, R subtalar**	L elbow, R wrist, 7 MCPs, **L ankle, L subtalar**	Neg
38	**L TMJ, R TMJ,** L shoulder, L elbow, 5 MCPs, **thumb CMC**	L elbow, **thumb CMC**, L knee	**L TMJ, R TMJ,** L shoulder, L elbow, 10 MCPs, **L thumb CMC, R thumb CMC,** L knee	Neg
41	Unknown	Unknown	Unknown	Pos
44	L shoulder, R shoulder, L wrist, R wrist, L ring MCP, R ring MCP, L little MCP, R little MCP	L ring MCP, R ring MCP, L little MCP, R little MCP	L shoulder, R shoulder, L wrist, R wrist, L ring MCP, R ring MCP, L little MCP, R little MCP, **R hip**	Neg
49	None	Unknown	Unknown	Neg
50	R wrist, L knee, **L ankle**	None	R wrist, L knee, **L ankle**	Neg
56	**L TMJ, R TMJ,** 2 finger joints	4 finger joints	**L TMJ, R TMJ,** 4 finger joints	Neg
58	L wrist, R wrist, 9 MCPs, 8 PIPs	L wrist, 10 MCPs, 10 PIPs	L wrist, R wrist, 10 MCPs, 10 PIPs	Pos

Joints not included within the DAS28 joint count are shown in bold.

CMC, carpometacarpal joint; L, left; MCP, metacarpophalangeal joint; Neg, negative; PIP, proximal interphalangeal joint; Pos, positive; R, right; RF, rheumatoid factor; TMJ, temporomandibular joint.

In many countries where DAS28 is used in adults with polyarthritis regardless of age of onset, this may have important implications when determining which patients qualify for biological drugs. These data support ongoing use of JADAS as the more appropriate disease activity measure to use in adults with polyJIA. A larger study to determine how this may impact on therapeutic decisions is warranted.
